# The impact of heterogeneous interpersonal relationships on promoting cooperation under the reputation mechanisms in public goods game

**DOI:** 10.1371/journal.pone.0332474

**Published:** 2025-09-25

**Authors:** Zhenghong Wu, Yining Mu, Zihan Wang

**Affiliations:** School of Economics and Management, Civil Aviation University of China, Tianjin, China; Teesside University, UNITED KINGDOM OF GREAT BRITAIN AND NORTHERN IRELAND

## Abstract

Although the principle of “case-by-case analysis” is widely endorsed, achieving complete rationality in the real world continues to be fraught with difficulties. Interpersonal relationships are heterogeneous, and the influence of social relationships and worldly wisdom on reputation evaluation should not be overlooked. Therefore, based on indirect reciprocity theory, this paper constructs a public goods game model with strategy update rules driven by reputation mechanism, aiming to investigate the impact of heterogeneous interpersonal relationships on the promotion of cooperative public goods provision among residents. The paper categorizes interpersonal relationships into three types, and proposes three corresponding reputation evaluation rules. Simulation results demonstrate that varying intensities of interpersonal relationships result in different levels of cooperation. When conducting public activities, externalities and the organizational efficiency of managers must be considered. Meanwhile, cooperation is difficult to sustain if the reputation mechanism fails to function effectively.

## Introduction

Public goods, including clean air, public parks, and national defense, bestow extensive societal benefits. Investigating how these goods are provided and maintained is essential for the well-being of communities and nations. Public goods are defined by non-excludability (no individual can be excluded from using them) and non-rivalry (one person’s consumption does not reduce another’s). These characteristics often result in the free-rider problem, where individuals consume the good without contributing to its provision. Therefore, allowing government to provide public goods is a suitable option [1–4]. However, government provision alone often results in low efficiency and a significant financial burden [5].

To address the insufficiency of government-provided public goods, some scholars have proposed a diversified provision model involving social organizations [6,7], enterprises [8], and residents [9]. This approach not only leverages market mechanisms for efficient resource allocation, but also alleviates the financial burden on the government [10–15]. Particularly with resident participation, provision can better align with demand and enhance public satisfaction with public goods [16]. Therefore, guiding residents to participate in the provision of public goods has become a key strategy for alleviating inefficiency and the fiscal burden. Due to the non-excludability and non-rivalry of public goods, free-riding becomes the optimal strategy, making it difficult for cooperative provision to be sustained among rational individuals. Moreover, Darwin’s theory of evolution also posits that selfish behavior tends to be more advantageous in competitive contexts [17]. In view of this, how to enable residents to cooperate sustainably in provision has become an urgent problem to be solved in the field of public goods provision.

Fortunately, individuals in the real world do not always act with complete rationality when making decisions. Cooperative behavior is prevalent, and individuals often coordinate their actions for the common good in daily life [18–22]. However, evolutionary game theory explains how cooperation evolves and is sustained in different environments [23,24]. Specifically, the public goods game (PGG) offers a robust theoretical framework for analyzing complex human social behavior and explaining the dynamics and barriers to cooperation [25–28]. At present, several mechanisms have been identified as promoting cooperative behavior, including kin selection [29,30], group selection [31,32], rewards [33–35], punishment [36–38], tolerance [39–41], heuristics [42–44], heterogeneity [45–47], direct reciprocity [48,49], and indirect reciprocity [50–52].

Most studies have shown that the reputation mechanism in indirect reciprocity is a key factor in promoting cooperation in PGG. In the context of the reputation mechanism, various factors have been identified that affect cooperative provision of public goods among residents, including visibility and observability of individual contributions [53], social norms [54], ethnic and social diversity [55], economic incentives and sanctions [34,38], historical factors [56], and behavioral traits [57,58]. Such as, Zhou et al., found that as the reputation factor increased, the level of facilitated cooperation also rose [59]. Quan et al., examined the relative and absolute value of reputation and its discount effect on strategic imitation [60]. Wang et al., proposed a novel tolerance-based reputation scoring mechanism based on historical donations and found that strict scoring rules could enhance cooperation in most cases [56]. Many other scholars have also investigated the role of reputation in promoting cooperation [61–66].

Furthermore, some scholars have started to focus on the impact of interpersonal relationships on reputation evaluation [67–69]. Previous studies have shown that fostering trust [57], facilitating the exchange of reputational information [70], and enhancing social bonds [71] can significantly improve cooperative behaviors driven by reputation systems through interpersonal interactions. However, most studies assumed that interpersonal relationships are homogeneous, with even fewer categorizing them based on their level of intimacy. Different types of interpersonal relationships involve varying levels of trust, communication frequency, and social bonds. In general, strong relationships can strengthen the role of reputation mechanisms in promoting cooperation through factors such as high trust levels [57,72]. It is also important to note that overly intimate relationships may lead to biased behavior, favoritism, or protectionism, which can undermine cooperation. Therefore, exploring the impact of heterogeneous interpersonal relationships on promoting cooperation within the context of reputation mechanisms is valuable. To achieve this, this paper extracts key elements of residents’ provision of public goods from real cases and constructs a group evolution model based on the PGG to explore the impact of varying degrees of interpersonal closeness on residents’ cooperative provision of public goods under the reputation mechanism. This paper findings offer valuable insights for organizers to guide residents in the cooperative provision of public goods across varying interpersonal relationship contexts.

The rest of this paper is structured as follows. The Model section presents the PGG model. The Simulation results and discussion section discusses the results of the simulations. The Conclusion and implication section concludes the paper with key findings, implications, and directions for future research.

## Model

### Problem description

Inspired by Hwang [73], this paper proposes reputation evaluation criteria based on three key types of interpersonal relationships: emotional relationship (ER), mixed relationship (MR), and instrumental relationship (IR). ER refers to a close, interdependent relationship typically observed between individuals such as spouses, parents and children, or close friends. MR is a relatively harmonious relationship between individuals who know each other and have regular interactions, such as ordinary friends, neighbors, classmates, or colleagues. IR refers to a social relationship where both parties engage in fair trade based on a specific purpose. In summary, the evaluator will assess reputation not only based on an individual’s contribution but also considering the heterogeneous interpersonal relationship between the parties involved.

This paper is inspired by village opera in Jiangsu and Zhejiang provinces, where funding primarily relies on villagers’ voluntary and unpaid donations. However, interpersonal relationships in villages are complex and heterogeneous, making it difficult for individuals to judge things according to situations. The influence of social relationships and worldly wisdom on residents’ reputation evaluations cannot be overlooked. Previous studies rarely considered the role of heterogeneous interpersonal relationships in PGG. Therefore, This paper investigates the impact of heterogeneous interpersonal relationships within the reputation mechanism on promoting residents’ cooperative provision of public goods. If there is an impact, how the scale and structure of interpersonal relationships in different contexts influence the level of cooperation and the role of the reputation mechanism in this process.

### Public goods game model

The PGG model is constructed on a scale-free network where individuals are randomly distributed. Each agent has a distinct number of neighbors, reflecting the heterogeneity of the real world. We assume that the average degree of the scale-free network is *k*, and each agent participates in a number of PGGs equal to the number of neighbors plus one. Initially, all participants select their strategies with equal probability, becoming either cooperators or defectors. Cooperators contribute one unit cost to the public pool (*c*_*i*_ = 1), while defectors contribute nothing (*c*_*i*_ = 0). Thus, the total payoff for each agent *i* is calculated, as shown in Equation (1):


$$P_{i}=\sum_{j\in \Omega_{i}}P_{i}^{j}=\sum_{j\in \Omega_{i}}\left(r\frac{c_{j}}{k_{j}+1}-c_{i}\right)$$ (1)

where *Ω*_*i*_ is the set of PGG groups in which agent *i* participates. *r is* synergy factor. *c*_*i*_ is the contribution of agent *i*, which is 0 or 1. *c*_*j*_ is total contribution of all cooperators in group j. *k*_*j*_ is the number of neighbors of agent *i* in group *j.*

### Reputation update rule

After the donation, the donation information is made public and serves as the basis for others to evaluate individual reputation. At step *t*, each individual’s reputation comprises two components: the previous round’s reputation *R*_*i*_(*t*-1) and the current evaluation based on donation behavior *∆R*_*i*_(*t*). The reputation update rule is given in Equation (2):


$$R_{i}(t)=R_{i}(t-1)+\Delta R_{i}(t)$$ (2)

where *∆R*_*i*_(*t*) is the sum of *R*_*iER*_(*t*), *R*_*iMR*_(*t*) and *R*_*iIR*_(*t*), which will be explained in detail in the following section.

### The rules of heterogeneous reputation evaluation

Each agent is initially assigned a random reputation value. Specifically, the initial reputation *R*_*i*_(0) of agent *i* is randomly drawn from the interval [0, 200]. If an agent’s reputation exceeds 200, it is capped at 200. Similarly, if it falls below 0, it is set to 0.

**Fig 1:**
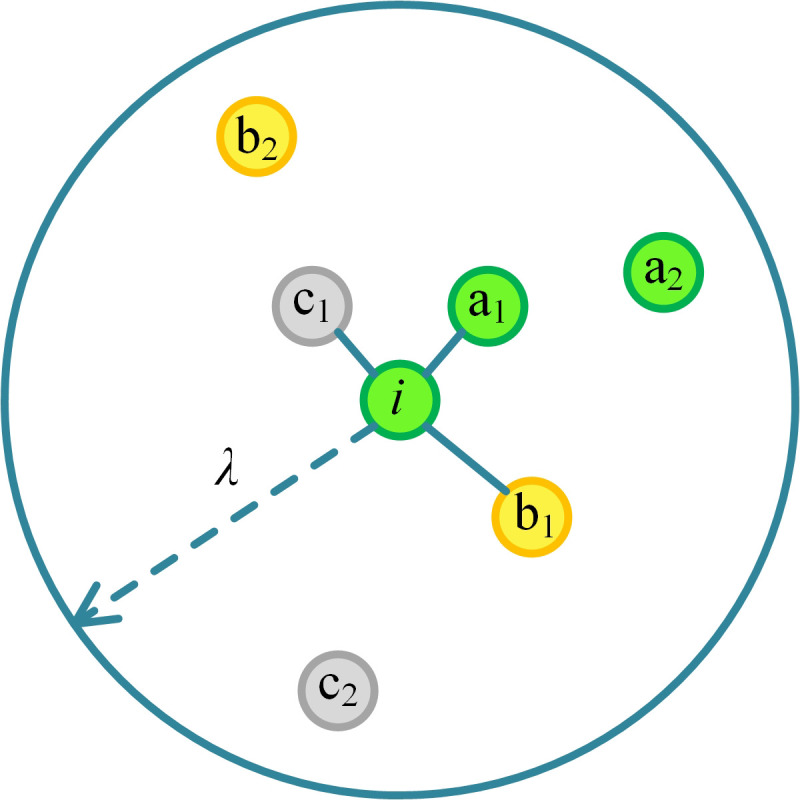
Ilustration of three types of interpersonal relationships. The outermost circle represents the reference circle with agent *i* as its center and *λ* as its radius. In the figure, colored nodes represent agents belonging to distinct emotional relationship (ER) subgroups, while gray nodes indicate agents with no ER connections. The color coding is used solely to visualize ER subgroup structure and does not imply spatial proximity or topological organization.

In this paper, a randomly selected agent *i* is assumed to possess a reference circle, centered at itself, with a radius *λ*, as illustrated in Fig 1, representing the scope of its social circle. Only evaluations from individuals within the social circle affect the reputation of agent *i.* For simplicity, it is assumed that all agents share reference circles of equal size in the simulation. Interpersonal relationships are classified into three types: ER, MR, and IR, each adopting distinct rules for reputation assessment. The three reputation evaluation rules are introduced below.

**Reputation evaluation rule of ER:** As is shown in Fig 1, the gray nodes indicate that they don’t have ERs with others, while the other colored nodes have. In reality, the former resemble ordinary individuals, while the latter resemble extended families with blood ties. Specifically, in the model, a stable relationship between individuals sharing the same color (except gray) within the social circle is defined as an ER, regardless of the presence of a link. For example, a_1_ and a_2_ both have emotional relationships with agent *i*. Under ER, participants will refrain from making negative judgments, even if agent *i* chooses to be a free rider. The ER formula for reputation evaluation as shown in Equation (3):


$$R_{iER}(t)=\{\;\;\;\begin{array}{c} {}*20c1c_{i}=1\\ 0c_{i}=0 \end{array}$$ (3)

where *R*_*iER*_(*t*) denotes the reputation evaluated by an individual within the ER of agent *i.*

**Reputation evaluation rule of MR:** Consider the green agent *i* in Fig 1 as an example. In the model, MR represents the interpersonal relationship between agent *i* and a link-neighbor of a different color, such as between agent *i* and b_1_, or between agent *i* and c1. The number of serial betrayals by agent *i* is considered when determining the individual’s reputation. If the number of betrayals is within the tolerance threshold *α*, the individual will tolerate agent *i*’s defection at step *t*. Accordingly, once it exceeds *α*, other participants will focus on agent *i*’s contribution and make negative judgments about the reputation. The MR formula for reputation evaluation as shown in Equation (4):


$$R_{iMR}(t)=\{\;\;\;\begin{array}{c} {}*20l1c_{i}=1\\ 0c_{i}=0,F\leq \alpha \\ -1c_{i}=0,F>\alpha \end{array}$$ (4)

where *R*_*iMR*_(*t*) indicates the reputation given by an individual following MR with agent *i. F* is the number of consecutive defections by agent *i*.

**Reputation evaluation rule of IR:** The IR represents the relationship between agent *i* and nodes that are neither connected to, nor share the same color as, agent *i* in the reference circle shown in Fig 1, such as between agent *i* and b_2_, or agent *i* and c_2_. If the two are in an IR, they evaluate each other’s reputation based on their contributions to the public pool. They think that contributions increase reputation, while defections decrease it. The IR formula for reputation evaluation as shown in Equation (5):


$$R_{iIR}(t)=\{\;\;\;\begin{array}{c} l1c_{i}=1\\ -1c_{i}=0 \end{array}$$ (5)

where *R*_*iIR*_(*t*) is the reputation evaluated by an individual conforming to IR with agent *i*.

### Strategy update rules based on reputation mechanism

Most individuals care about face, which is represented by reputation in this paper. If an individual receives numerous negative evaluations, causing the reputation to fall below the threshold *R*_*T*_, embarrassment and shame will result [53]. Therefore, when *R*_*i*_(*t*)<*R*_*T*_, the agent must contribute in the next step to improve the reputation and save face. Conversely, when *R*_*i*_(*t*)≥*R*_*T*_, individuals follow *t*he principle of primacy of returns, aiming to optimize their returns by randomly copying a neighbor’s strategy from the previous round with a given probability, as shown in Equation (6):


$$P(S_{i}\leftarrow S_{j})=\frac{1}{1+\exp[(p_{i}-p_{j})/\kappa ]}$$ (6)

where *κ* is the noise figure, representing the uncertainty in the participant’s strategy choice. *S*_*i*_ and *S*_*j*_ represent strategies of agent *i* and *j*, respectively. *p*_*i*_ and *p*_*j*_ refer to the total payoffs of agent *i* and *j*, respectively.

## Simulation results and discussion

Although ER, MR, and IR relationships commonly coexist in real-world networks, we adopt an experimental control strategy in our simulations to isolate the marginal influence of each relationship type on cooperative dynamics. Specifically, we systematically vary the parameters of one relationship type at a time while keeping the others constant, in order to evaluate their respective effects on cooperation.

To minimize stochastic errors in Monte Carlo simulation (MCS), multiple independent simulations were performed to investigate evolutionary dynamics on a scale-free network. Initially, 10% of individuals are assigned ERs and randomly grouped into three groups (*g*) to reflect realistic conditions. The proportion of cooperation (*ρ*_*c*_) is set to 0.5. The other key parameters are set as follows: N = 200, *k* = 4, *r* = 2, *λ* = 6, *α* = 2, *κ* = 0.1, *R*_*T*_ = 20. All results are calculated by 20 independent simulations within a total of 10000 steps. Each data point is calculated by averaging the cooperation proportion over the last 1,000 steps after the system reaches equilibrium. The parameter Settings are shown in Table 1.

**Table 1:** Parameters setting.

Parameters	Notes
N	Total number of agents
*k*	Average number of neighbors
*r*	Synergy factor
*λ*	The radius of the agent’s reference circle
*α*	Tolerance threshold in mixed relationships
*κ*	The noise figure
*R*_*T*_	Reputation threshold of the agent
*ρ*_*E*_	The intensity of affective relationships
*g*	The number of small groups within ER groups
*ρ*_*c*_	The proportion of cooperation

To improve the clarity of the model design, we provide additional explanations for several key parameters listed in Table 1:

*ρ*_*E*_ (emotional relationship intensity): This parameter denotes the proportion of agents involved in emotional relationships (ERs) within the network. Specifically, *ρ*_*E*_ × *N* agents are randomly assigned as ER members and further divided into subgroups to form stable emotional clusters.
*g* (number of emotional subgroups): This denotes the number of subgroups into which ER agents are partitioned. All ER-designated agents are evenly allocated to *g* subgroups, simulating multiple “families” or “close-knit social circles.” Emotional ties are assumed to exist only within each subgroup; no ER connections are assumed between agents from different subgroups.
*λ* (social radius): This parameter defines the range within which an agent can receive reputation information. In the scale-free network used in our simulations, node distance is measured by the number of hops (i.e., shortest path length), rather than by geometric distance. An agent *i* receives reputation evaluations only from nodes whose shortest path distance to *i* is less than or equal to *λ*. Thus, a larger *λ* expands the agent’s reputation horizon, increasing the effective influence range of the reputation mechanism.

### The impact of emotional relationships

In the experiment assessing the impact of ER on cooperation, we fixed the number of ER agents (*ρ*_*E*_ > 0) and maintained the influence mechanisms of MR and IR relationships at their default settings, without targeted manipulation. This setup allowed us to isolate the marginal effects of ER intensity and intra-group structure on cooperative behavior.

**Fig 2:**
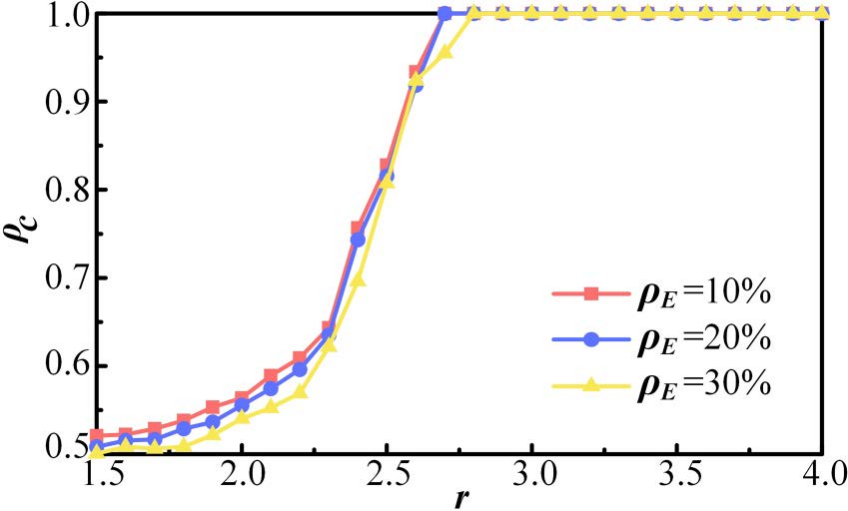
The proportion of cooperation ρ_*c*_ as a function of synergy factor *r* for different values of ρ_*E*_. Other parameters are set as follows: *k* = 4, *λ* = 6, *α* = 2, *κ* = 0.1, and *R*_*T*_ = 20.

First, the effect of the individual aggregate with ER on the evolution of cooperation is investigated. Fig 2 illustrates the proportion of cooperation *ρ*_*c*_ as a function of synergy factor *r* for different values of *ρ*_*E*_. As shown in Fig 2, variations in *ρ*_*E*_ influence the proportion of cooperation *ρ*_*c*_, and this effect is constrained by the synergy factor *r*. That is, there exists a critical threshold *r’*. When *r* < *r’*, the proportion of cooperation *ρ*_*c*_ decreases as *ρ*_*E*_ increases. When *r* ≥ *r’*, the proportion of cooperation *ρ*_*c*_ is not affected by the change of *ρ*_*E*_. According to Fig 2, when *r* < 2.7, the proportion of cooperation *ρ*_*c*_ declines with increasing *ρ*_*E*_. When *ρ*_*E*_ is 10%, it provides only a slight improvement in the overall proportion of cooperation. For *r* > 2.7, further increases in the synergy factor *r* result in no significant change the proportion of cooperation in the equilibrium.

A larger value of *ρ*_*E*_ implies that more individuals will shield non-cooperative agents. A smaller value of *ρ*_*E*_ suggests that most individuals act independently, leading to reduced favoritism within the system. Greater intimacy in relationships within a society fosters more favoritism and protective behavior, thereby hindering cooperation efficiency. The results also suggest that the nature of the activity or project should be considered when organizing a public event.

Next, the impact of heterogeneity in the distribution within the ER population on the evolution of cooperation is investigated. The parameter *g* is introduced to represent the number of groups with distinct affective relationships within the population. For example, in a village, individuals a1 to a10 belong to one family, while b1 to b10 belong to another. As *g* increases, the number of groups with distinct affective types in the village increases, and conversely, this number reduces.

**Fig 3:**
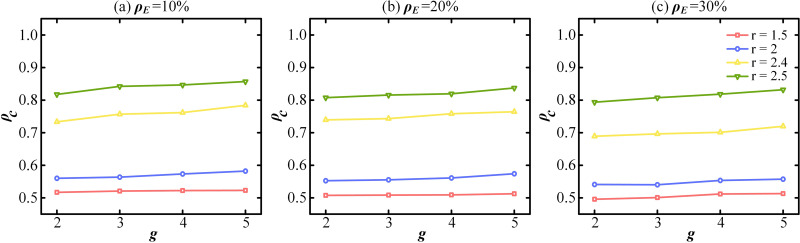
The proportion of cooperation *ρ*_*c*_ as the internal structure of the group with ER changes for different values of r. Here, *ρ*_*E*_ = 10% in (a), *ρ*_*E*_ = 20% in (b) and *ρ*_*E*_ = 30% in (c).

The relationship between the parameter *g* and the proportion of cooperation *ρ*_*c*_ is presented in Fig 3. Figs 3(a), (b) and (c) correspond to *ρ*_*E*_ = 10%, *ρ*_*E*_ = 20% and *ρ*_*E*_ = 30%, respectively. The curves are generated under different synergy factors: *r* = 1.5, 2, 2.4 and 2.5. In Fig 3(a), the cooperative evolution results are basically similar. A similar trend is observed in Figs 3(b) and (c). These findings indicate that variations in *g* exert minimal influence on the proportion of cooperation. Additionally, the results validate the robustness of the model against changes in group number. Consequently, *g* = 3 is adopted in subsequent analysis, as it does not significantly affect the final outcomes.

### The impact of mixed relationships

In the experiment investigating the effect of MR on cooperation, we varied the network’s average degree (*k*) to modulate MR intensity. According to the model design, an MR exists between two nodes if they are directly connected (i.e., neighbors) but do not share an ER. In this set of simulations, the proportion of ER agents was held constant. As a result, variations in *k* did not alter the absolute number of ER agents or generate new ER ties. Instead, increasing *k* led to more non-ER neighbors, thereby enhancing both the quantity and density of MR connections in the network.

**Fig 4:**
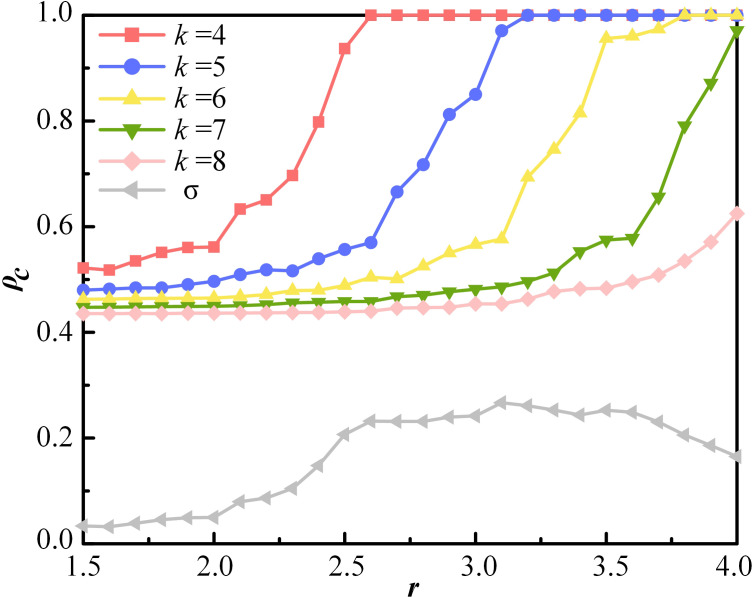
The proportion of cooperation *ρ*_*c*_ as a function of synergy factor *r* for different values of k. Other parameters are set as follows, *ρ*_*E*_ = 10%, *λ* = 6, *α* = 2, *κ* = 0.1, *R*_*T*_ = 20.

MR connections refer to interpersonal links between agents who are connected but not involved in ERs. To examine the effect of MR intensity on cooperation, the average number of neighbors *k* is varied. The evolutionary results are illustrated in Fig 4 for network average degrees of 4, 5, 6, 7, and 8. In general, MR intensity exhibits a negative correlation with *ρ*_*c*_, that is, lower values of *k* tend to yield higher levels of cooperation. As observed in Fig 4, the red curve representing *k* = 4 lies above the other curves in most cases. However, the negative correlation between MR intensity and *ρ*_*c*_ is affected by *r*, and it can be seen from Fig 4 that all lines show a trend of convergence, divergence and re-convergence. To further quantify the influence of *r*, the standard deviation σ of *ρ*_*c*_ is computed, revealing a rise-and-fall pattern.

In general, the increase of MR is not conducive to the cooperative behavior of the group. This occurs because higher MR intensity fosters concealment within the group. In such social circles, a greater number of individuals are willing to tolerate defection by agent *i*, leading to a decrease in the proportion of cooperation. From the perspective of network structure, an influential individual in a social circle resembles a core node in a scale-free network. Core nodes are highly connected and exert dominant influence within the network. Once a central individual chooses to defect, more neighbors will follow this behavior with a certain probability. In summary, the number of cooperators declines with increasing MR intensity at equilibrium. Furthermore, higher externalities in cooperative provision encourage greater participation in cooperation. Within the scope of this study, the synergy factor *r* exhibits a trend where differences in cooperation across various *k* values initially increase and then decrease. This indicates that as *r* increases, the disparity in the proportion of cooperation under different MR intensities first widens, but narrows after reaching a certain threshold. This dynamic is further quantified by calculating the standard deviation σ of *ρ*_*c*_, which exhibits a rising and then falling trend as *r* increases, reflecting the changing influence of the synergy factor on cooperation differences.

**Fig 5:**
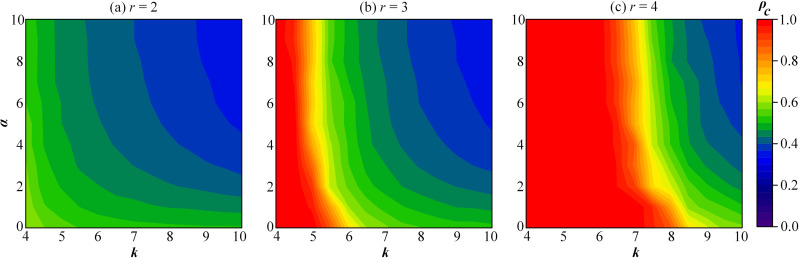
The contour map of the proportion of cooperation ρ_*c*_ as a function of average degree of network *k* and tolerance threshold α. Here, *r* = 2 in panel (a), *r* = 3 in panel (b) and *r* = 4 in panel (c).

Furthermore, the influence of the tolerance threshold *α* on individual strategic choice in MR is examined through simulations. Fig 5 depicted how the average number of neighbors *k* and the tolerance threshold *α* affect the proportion of cooperators in the group. In Fig 5(a), with *k* held constant, the cooperation rate decrease as *α* increases. As the synergy factor *r* increases, the effective range of *α* narrows, as reflected by the expansion of the red region. Specifically, in Figs 5 (b) and (c), when *k* is less than approximately 5 and 7, respectively, individual strategic choices are insensitive to the tolerance threshold *α*. For instance, in Fig 5(b), when *k* = 4, a high level of cooperation is maintained regardless of the value of *α*. By contrast, for larger values of *k* becomes more pronounced in the evolution of cooperation, as shown in Figs 5 (b) and (c).

In social networks with a high proportion of MR, individuals expect future reciprocation. As a result, relationships tend to be preserved rather than reputations tarnished by defection within tolerance limits. This often leads to a “favor trap” within the organization. Additionally, as shown in Fig 5, a fully cooperative state can only be achieved under high *α* and large *r*. However, such conditions are relatively rare in public activities with significant externalities. Therefore, it is necessary to guide individuals to reduce their tolerance levels to better accommodate more practical scenarios.

**Fig 6:**
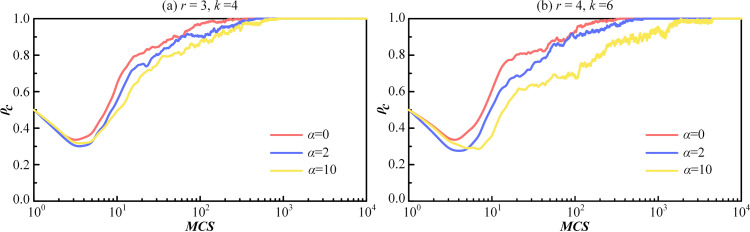
The proportion of cooperation ρ_*c*_ changes over time for different parameter combinations at each MCS time step. (a) Under three different tolerance thresholds *α* for fixed synergy factor *r* = 3 and average degree *k* = 4. (b) Under three different tolerance thresholds *α* for fixed synergy factor *r* = 4 and average degree *k* = 6.

To further investigate the influence of the tolerance threshold *α* on the evolution of cooperation, Fig 6 illustrates the evolutionary process of cooperation proportion over time for two different parameter sets. In Fig 6, *r* = 3, *k* = 4 in Fig 6 (a) and *r* = 4, *k* = 6 in Fig 6 (b) correspond to the critical values shown in Figs 5(b) and (c), respectively. Clearly, all curves clearly decrease initially and then increase, eventually reaching equilibrium. At the same time, as the tolerance threshold *α* increases, the curves reaching the fully cooperative state shift to the right. It is worth noting that although there is no significant difference in the final evolutionary result, the defection behavior cannot be blindly tolerated in the social system. As society develops, As society develops, MR, which emphasizes emotional ties and social connections, will gradually become dominant and widespread. Therefore, efforts are required to eliminate indulgent behavior and actively promote the emergence of cooperation.

### The impact of instrumental relationships

In the experiment assessing the effect of instrumental relationships (IR) on cooperation, we primarily varied the social radius (*λ*) to control each agent’s interaction neighborhood, thereby simulating the expansion of the reputation evaluation scope linked to IR. The intensities of emotional (ER) and mixed (MR) relationships were held constant to isolate the specific effect of IR on cooperative behavior.

**Fig 7:**
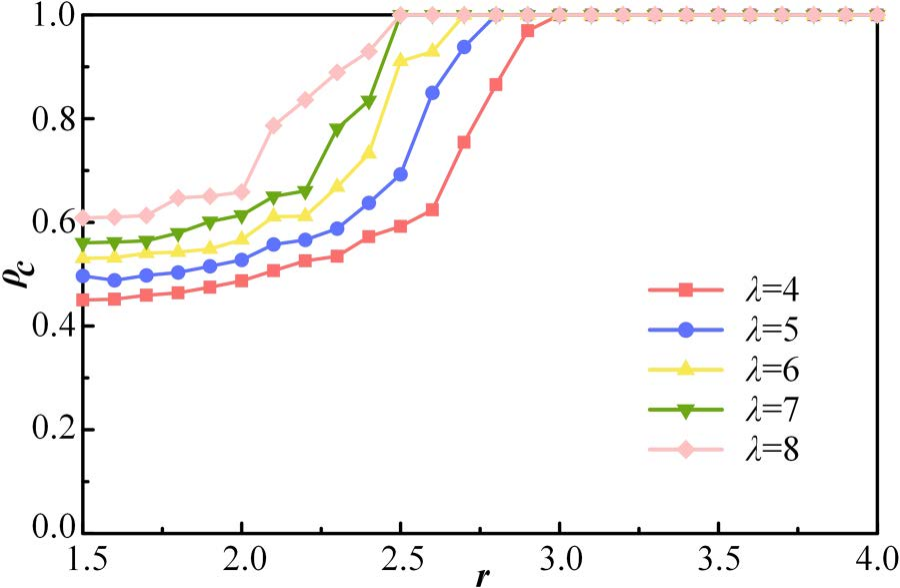
The proportion of cooperation *ρ*_*c*_ as a function of *λ* with the variation of synergy factor r. Other parameters are set as follows, *ρ*_*E*_ = 10%, *k* = 4, *α* = 2, *κ* = 0.1, *R*_*T*_ = 20.

The impact of IR intensity in interpersonal relationships on the evolution of cooperation is subsequently examined. In real life, most interpersonal connections are relatively weak and distant, characterized as IR. To represent increased IR intensity, the agents’ reference radius *λ* is enlarged. Fig 7 illustrates the effect of *λ* when other parameters are constant expect the synergy factor *r*. Overall, all the curves exhibit a similar trend and appear to have a critical value *r*. For example, when *λ* = 4, the proportion of cooperation remains relatively stable for *r* < 2, followed by a sharp increase. The curve then increases more rapidly until full cooperation is achieved. After *r = *3, the proportion of cooperation is not affected by *λ* when the system achieves equilibrium. However, for *r* < 3, variations in *λ* significantly influence the evolution of cooperation. A larger *λ* more effectively suppresses defection within the group.

It can be deduced that the larger *λ* is, the greater the proportion of IR in social interactions, which makes individuals’ reputations more reliant on rational judgments based on contributions. Individuals in IR pay more attention to facts. Reputation is more widely evaluated based on actions rather than interpersonal connections. Undeniably, an increase in spontaneous rational decision-making is advantageous for the emergence of cooperators. Although achieving a purely rule-based society in real life is challenging, fairness in the process must be emphasized to promote the development of social civilization.

### Reputation mechanism analysis

**Fig 8:**
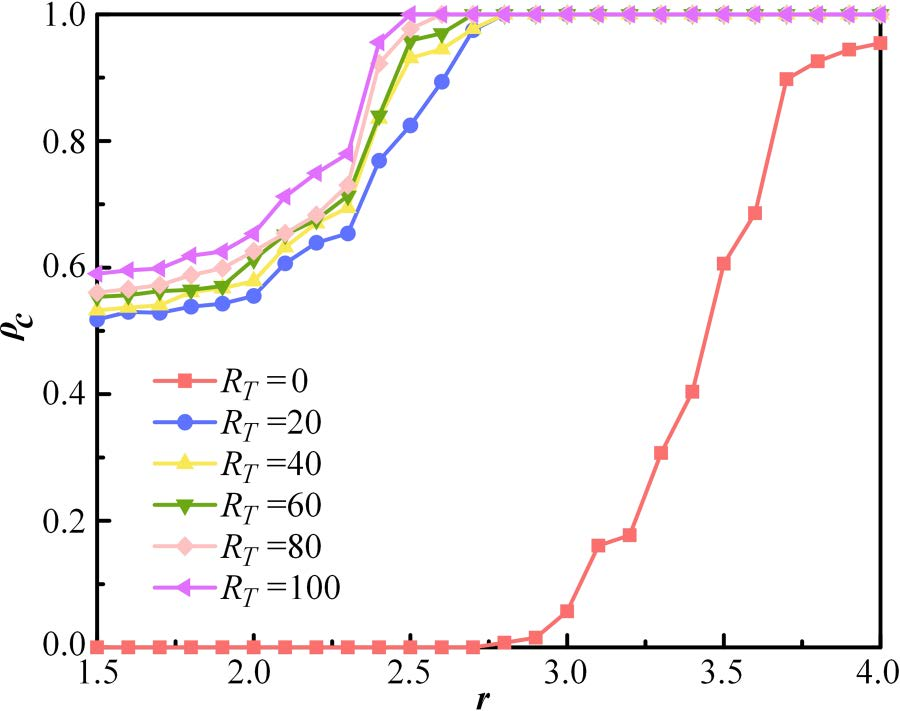
The proportion of cooperation *ρ*_*c*_ as a function of synergy factor *r* for different values of *R*_*T*_. Other parameters are set as follows, *ρ*_*E*_ = 10%, *k* = 4, *λ* = 6, *α* = 2, *κ* = 0.1.

The above analysis mainly discuss the degree of tolerance towards others in interpersonal interactions. However, it is essential to emphasize the role of self-discipline in social cooperation, specifically the reputation threshold *R*_*T*_ addressed in this paper. The effect of the reputation threshold *R*_*T*_ on the proportion of cooperation *ρ*_*c*_ under different synergy factors *r* is further examined, as shown in Fig 8. In this paper, the reputation accumulated through an agent’s contributions is used to represent “face”, a concept highly valued in society. When *R*_*T*_ ≠ 0, the curves exhibit similar trends. It is obvious that *ρ*_*c*_ increases as *R*_*T*_ enlarges. Notably, when *R*_*T*_ = 0 and *r* < 2.8, the cooperation rate remains zero. A small number of cooperators appear when *r* = 2.8.

The simulation results indicate that when *R*_*T*_ ≠ 0, maintaining face is conducive to promoting cooperation. That is, when individuals are sensitive to others’ evaluations, they are motivated to contribute to maintain a good reputation. The higher *R*_*T*_ is, the more concerned individuals become with their image and status in social interactions. Additionally, the phenomenon when *R*_*T*_ = 0 illustrates that maintaining cooperation is challenging if individuals ignore external institutional norms, rendering the reputation mechanism ineffective.

**Fig 9:**
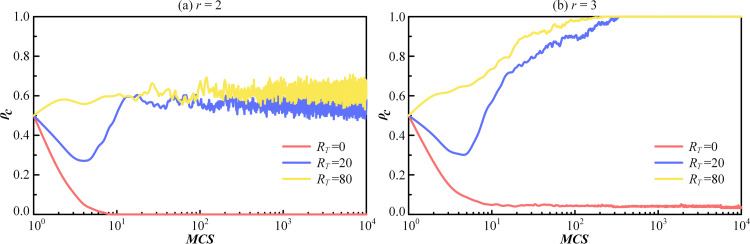
The time evolution of the proportion of cooperation ρc for R_T_ = 0, 20 and 80. The synergy factor r is set to be 2 in (a) and 3 in (b).

To further explore the role of the reputation mechanism in PGG, the dynamic evolution of the cooperation proportion is plotted at each MCS step for different values of *r* and *R*_*T*_. The reputation threshold *R*_*T*_ is set to 0, 20 and 80. The evolutionary curves are shown in Figs 9(a) and (b), corresponding to *r* = 2 and *r* = 3, respectively. It can be observed that when RT = 0, the proportion of cooperation decreases monotonically at the beginning of the evolution until to 0 or a minimal value. When *R*_*T*_ = 20, the proportion of cooperation initially decreases and then gradually increases around step 5. For *R*_*T*_ = 80, the proportion of cooperation initially increases. As shown in Fig 9(b), the curve for *R*_*T*_ = 80 continues to rise, eventually reaching full cooperation. Consequently, higher *R*_*T*_ leads to more cooperative behaviors in the early stages. In contrast, when *R*_*T*_ is low, individuals are motivated by self-interest and tend to defect. The result suggests that cooperative behavior emerges and eventually stabilizes at equilibrium under the influence of the reputation mechanism.

### Robustness of the model

**Fig 10:**
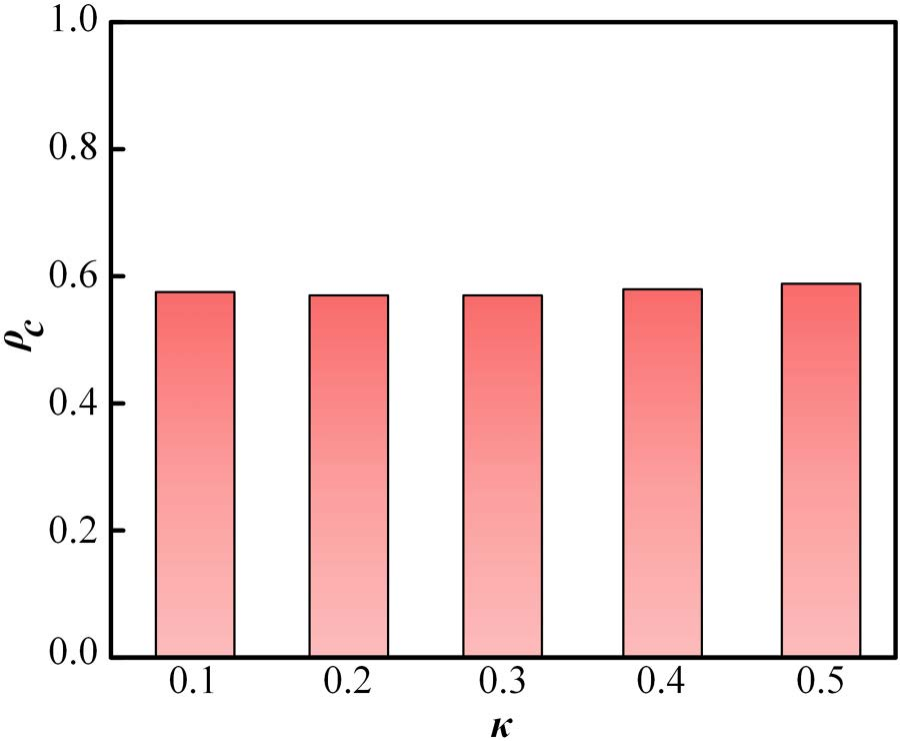
The bar graph of the proportion of cooperation ρc with noise figure κ set at 0.1,0.2,0.3,0.4 and 0.5.

Finally, to validate the robustness of the model, Fig 10 depicts the relationship between the proportion of cooperation *ρ*_*c*_ and the noise figure *κ*. As observed, the proportion of cooperation remains relatively stable across different values of *κ*. Specifically, *ρ*_*c*_ reaches a maximum of 0.584 at *κ* = 0.5, and a minimum of 0.570 at *κ* = 0.2. The fluctuation range of *ρ*_*c*_ does not exceed 0.02, confirming the robustness of the proposed model.

## Conclusion and implication

### Conclusions

Given the influence of social relationships and worldly wisdom, it is often difficult to judge the facts from the facts. Based on existing research, this paper classifies interpersonal relationships into three types to examine how heterogeneous relationships under the reputation mechanism affect cooperation in PGG. Through simulation experiment and analysis, the following conclusions are drawn.

(ⅰ) Changes in the distribution structure within the ER group have minimal impact on the proportion of cooperation. Furthermore, a smaller *ρ*_*E*_ promotes cooperation provision.
(ⅱ) An increase in MR reduces the proportion of cooperation. The tolerance threshold *α* positively influences cooperation, but its effective range diminishes as r increases.
(ⅲ) Increasing IR helps suppress defection under the reputation mechanism, allowing individuals’ reputations to be based more on rational evaluations of contributions.
(ⅳ) Without a functioning reputation mechanism, maintaining cooperation is challenging. High *R*_*T*_ levels promote the diffusion of cooperative behavior in public goods.

## Implications and future research

### Theoretical implications

(ⅰ) Studying residents’ cooperative provision of public goods from the perspective of bounded rationality offers a more realistic framework. Although Darwin’s evolutionary theory emphasizes competition as the norm, the rational man hypothesis suggests that non-excludability leads to free-riding, rendering voluntary cooperation by residents unsustainable. However, cooperative behaviors—such as subsidizing disadvantaged children and donating blood without compensation—are prevalent in real-world societies. The village opera studied here is a typical example. Findings based on the village opera further support the rationale for exploring how to guide residents in providing public goods under bounded rationality, consistent with the principles of behavioral economics.
(ⅱ) This paper incorporates varying intensities of interpersonal relationships, thereby broadening the scope of research on residents’ cooperative provision of public goods. At the same time, the results confirm that heterogeneous interpersonal relationships significantly affect cooperation, as different relationship types yield varying impacts on residents’ participation.
(ⅲ) The positive externality associated with cooperative provision of public goods is an important factor in encouraging residents’ cooperation. This study shows that the synergy factor *r* has a significant impact on the proportion of cooperation, with its range of variation significantly affecting the influence of other parameters on cooperation. Synergy factor *r* can be regarded as the positive externality of cooperative provision of public goods. As *r* increases, individuals benefit more per capita by choosing to cooperate. While this paper emphasizes cooperation from a bounded rationality perspective, it acknowledges the significant role of rational payoffs in fostering cooperation. Future research should classify public goods according to the positive externality of their cooperative provision and further advance studies to promote resident cooperation in public goods provision.

### Practical implications

(ⅰ) Analyzing promotional effects under varying scenarios helps organizers select appropriate public goods. This paper demonstrates that interpersonal relationship intensity, reputation threshold *R*_*T*_, and synergy factor *r* influence residents’ cooperative provision of public goods, providing a quantitative relationship based on village opera. This offers strategic insights for organizers to effectively guide cooperation and ensure sustainability.
(ⅱ) To improve residents’ cooperative provision, it is essential to actively promote social morality and ethics, as civilization should not equate to blind tolerance. The study finds that *α* and *R*_*T*_, representing heteronomy and self-discipline, respectively, are positively correlated with the proportion of cooperation. In other words, higher parameter values correspond to a higher proportion of cooperation. The study concludes that the government should actively publicize positive initiatives, enforce moral and ethical constraints, and avoid excessive tolerance of gray-area behaviors that, while not illegal, are immoral.
(ⅲ) The resident self-provision pattern is an effective way to reduce the government’s financial burden and improve the alignment of supply and demand. This study demonstrates, based on real cases, the feasibility of mobilizing residents to cooperate in public goods provision from a bounded rationality perspective. On one hand, residents raise funds to provide public goods, reducing the government’s fiscal expenditure. On the other hand, as consumers of public goods, residents become suppliers after self-provision, effectively bridging the supply and demand sides. Therefore, resident self-provision not only reduces the government’s financial burden but also improves the alignment of public goods supply and demand, ultimately enhancing residents’ satisfaction.

## Future research

Although the current model classifies interpersonal relationships into fixed categories—emotional (ER), mixed (MR), and instrumental (IR) ties—real-world relationships are often dynamic and evolve through repeated interactions. Emotional bonds may strengthen or weaken, instrumental ties can evolve into trust-based relationships, and reputation assessments adjust accordingly. Future research should consider co-evolutionary dynamics among social ties, strategic behaviors, and reputation updates, enabling relationship types to shift based on behavioral history and mutual feedback.

Recent developments in evolutionary game theory highlight the effectiveness of adaptive feedback mechanisms in promoting cooperation in social dilemmas. For example, adaptive rewards or penalties responsive to population-level cooperation have been shown to produce stable coexistence or evolutionary cycles [74,75]. Similarly, studies show that in collective-risk games, risk levels and social behavior mutually influence each other, with feedback loops playing a key role in shaping cooperation outcomes [76]. These findings suggest that incorporating feedback-driven mechanisms into interpersonal and reputational dynamics could yield a more realistic framework for modeling cooperation.

Furthermore, the study by Krellner M et al. emphasizes the role of joint commitments in reinforcing cooperation through indirect reciprocity [77]. This supports our findings, indicating that reputation systems embedded in relationships—whether based on interpersonal types or shared commitments—can serve as informal enforcement mechanisms in the absence of formal institutions. Future extensions of our model could investigate how public commitments, trust-building behaviors, and repeated cooperation signals reshape relationship structures and reputation thresholds over time.

Collectively, these directions suggest a more integrative modeling framework that captures the co-adaptation of individual behaviors, reputational dynamics, and social structures—an essential step toward understanding long-term cooperation in complex social systems.
